# Neuroprotection with Bioactive Compounds

**DOI:** 10.3390/nu15214612

**Published:** 2023-10-30

**Authors:** Carmen del Río, Antonio Segura-Carretero

**Affiliations:** 1 Institute of Biomedicine of Seville (IBiS), Hospital Universitario Virgen del Rocío, CSIC, Universidad de Sevilla, 41013 Seville, Spain; 2 Department of Analytical Chemistry, University of Granada, Av. Fuentenueva s/n, 18071 Granada, Spain; ansegura@ugr.es

Bioactive compounds are found in foods in small quantities and represent extra nutritional constituents known to exert beneficial effects on health beyond their nutritional value. They are produced as secondary metabolites in vegetables, fruit, and whole grains, but bioactive compounds are also found in fungi, animals, and bacteria. A wide variety of bioactive compounds are grouped according to their chemical structure. Some examples include polyphenols, triterpenes, phytosterols, polysaccharides, capsaicinoids, carotenoids, and tocopherols, among others [1].

Bioactive compounds have been used for multiple applications, including bioremediation (coagulants and biofilms), the food industry (by-products revalorisation), and the pharmaceutical sector. Given the increasing demand for healthier and natural food, the global market of bioactive ingredients is expected to rise in the upcoming years [1].

Accordingly, the interest in dietary approaches for the prevention of neurodegeneration has increased, and plant-derived bioactive compounds have drawn more attention in recent years due to their medicinal properties. Bioactive compounds can modulate several metabolic processes and promote better health conditions. They can directly modulate redox signalling [2] and influence epigenetic changes that affect DNA repair and expression [3]. Moreover, they have been reported to exhibit antioxidant and anti-inflammatory properties [2], as well as anti-aging effects [4]. Therefore, the beneficial effect of bioactive compounds on brain health needs to be better explored.

This Special Issue aims to gather new advances achieved in the association of isolated bioactive compounds and the phytochemical profile of plants with neuroprotection.

Some studies have focused on the neuroprotective potential of specific plant species. The beneficial health effects of the phytochemical composition of *Euterpe Genus* were reviewed, focusing on neuroprotection. The fruits of different Euterpe species are rich in phenolic acids and anthocyanins and have been shown to not only exert neuroprotection via antioxidant and anti-inflammatory molecular mechanisms but also by regulating neurotransmission and protein aggregation [5]. Another study focused on the therapeutic potential of the polyphenols present in the halophyte *Salicornia ramosissima* to design nutritional interventions for brain ischemia. Preclinical studies analysing isolated phenolic acids and flavonoids for their impact on preventing neuronal loss, blood–brain barrier (BBB) maintenance, and functional and cognitive improvements after ischemia were summarised. Mechanisms involved in these effects not only included antioxidant, anti-inflammatory, and antiapoptotic effects but also indirect actions on gut microbiota. The authors highlight the future possibilities of *S. ramosissima* polyphenol-rich extracts as a source of bioactive compounds for the nutraceutical industry [6]. In addition, an original article by Katayama et al. studied the beneficial effects of Fagopyrum esculentum Moench (buckwheat) on cognitive performance using a senescence-accelerated mouse model. Buckwheat is characterised by high amounts of fibre, proteins, polyphenols, minerals, and starch (amylose and amylopectin). Buckwheat and buckwheat starch-containing diets increased the expression levels of brain-derived neurotrophic factor (BDNF), preserved hippocampal neurons, and increased the diversity of gut microbiota, which may contribute to the prevention of age-related cognitive decline [7].

The benefits of gut microbiota regulation by bioactive compounds on brain health were also addressed by St-Laurent et al. Ginseng root is frequently used in traditional Chinese medicine, but the berries of this plant are regarded as a by-product. However, berries usually have higher concentrations of anti-depressant compounds. Ginsenosides and polysaccharides present in Ginseng berries have antioxidant effects, preserve BDNF levels, and regulate neurotransmitters, and their receptors to produce mental health benefits. In this review work, the authors emphasise that ginseng could promote mental health indirectly via the gut–brain axis [8].

As observed in Ginseng berries, different food by-products, such as seed, leaf, peel, pulp, or root, have emerged as sources of phytochemicals. In this line, the phenolic richness of fruit and vegetable by-products and their possible application as neuroprotectants were reviewed. The authors summarised the effect of polyphenol-rich by-product extracts in in vitro and in vivo models of neurodegeneration, with a special focus on their mechanism of action against oxidative stress, inflammation, excitotoxicity, and misfolded protein aggregation in Alzheimer’s and Parkinson’s disease conditions [9]. Polyphenols are among the best-studied bioactive compounds for neuroprotection. Accordingly, their role in the prevention and development of neurodegenerative disease was explored, focusing on their role in brain function (plasticity, cognition and mood modulation). The authors pointed out the main factors that limit their therapeutic efficacy, such as absorption or BBB penetration and the new strategies for improving the delivery [10].

A polyphenol-rich extract from *S. ramossisima* was studied for the first time in brain ischemia. The phytochemical composition of the extract was characterised by a significant abundance of caffeoylquinic acid derivatives, flavonoids and fatty acids. Dietary supplementation with the extract prevented hypoxia-induced death in flies and was shown to prevent ischemic-associated damage in mice, including brain infarct area and a systemic effect in the reduction in plasma oxidative stress markers [11]. Another study focused on neurovascular disease using a haemorrhage-induced brain damage model in rat pups. Treatment of immature rats with betulinic acid hydroxamate, a triterpenoid derivative designed to improve its pharmacological performance, improved motor and cognitive impairment and reduced long-term white matter injury by reducing oxidative stress, inflammation and excitotoxicity [12].

Other microvascular complications such as diabetic peripheral neuropathy may also benefit from the effect of bioactive compounds. Peripheral nerves can also be damaged due to ischemia secondary to a reduced capillary density. A meta-analysis of randomised clinical trials on the oral administration of alpha-lipoic acid determined a reduction in total symptom score and sensory symptoms, suggesting a potential use of alpha-lipoic acid in preventing neurological symptoms associated with diabetic peripheral neuropathy [13]. In this sense, a clinical study was conducted to identify cellular pathways elicited by polyunsaturated fatty acid (PUFA) supplementation in patients suffering from painful diabetic neuropathy. PUFAs are the most common bioactive lipids, among which we can find docosahexaenoic acid (DHA) and eicosapentaenoic acid (EPA). Daily supplementation with DHA-enriched capsules for 3 months resulted in metabolic changes towards a more anti-inflammatory state and increased membrane fluidity, reducing adverse symptoms associated with painful neuropathy [14].

Another class of bioactive compounds, dichloroacetic acid, was studied for the treatment of seizures. This organic acid, in combination with pyruvate, which is a substrate for energy production, was shown to reduce oxidative stress after seizures, reduce inflammatory cell activation in the brain and prevent neuronal death and cognitive impairment in a rat model of pilocarpine-induced seizure [15].

Overall, the aforementioned manuscripts encompass the health benefits of individual and combined bioactive compounds found in plants in relation to neuroprotection in a wide range of brain health conditions, including common neurodegenerative disorders, seizures, neurovascular diseases, age-related cognitive decline and mental health.
